# Trio-based whole exome sequencing in patients with ectopic posterior pituitary

**DOI:** 10.3389/fped.2024.1334610

**Published:** 2024-08-02

**Authors:** Arthur Lyra, Itatiana Ferreira Rodart, Lara Barros, Tatiane Sousa e Silva, Antônio José da Rocha, Cristiane Kochi, Carlos Alberto Longui

**Affiliations:** ^1^ Pediatric Endocrinology Unit, Pediatric Department, Irmandade da Santa Casa de Misericórdia de São Paulo and Santa Casa de Sao Paulo School of Medical Sciences, São Paulo, Brazil; ^2^ Department of Radiology, Irmandade da Santa Casa de Misericórdia de São Paulo and Santa Casa de Sao Paulo School of Medical Sciences, São Paulo, Brazil

**Keywords:** ectopic posterior pituitary, exome sequencing, pituitary stalk interruption syndrome, hypopituitarism, midline defects

## Abstract

**Introduction:**

Ectopic posterior pituitary (EPP) is a rare congenital abnormality, sometimes associated with other midline defects, such as pituitary stalk interruption syndrome (PSIS), in which thin or absent pituitary stalk and anterior pituitary hypoplasia are combined to EPP. Most cases are sporadic, with few reports of familial cases, and many congenital hypopituitarism (CH) cases remain unsolved.

**Objective:**

To search for candidate genes associated with this condition, we performed trio-based whole-exome sequencing (WES) on patients with EPP, including two familial cases.

**Methods:**

This study included subjects with EPP and PSIS diagnosed by a simple MRI protocol (FAST1.2). We performed two distinct analyses in the trio-based WES. We looked for previously described genes associated with pituitary development. Next, we investigated the whole exome for variants inherited in a pattern consistent with a monogenic etiology.

**Results:**

Ten families were evaluated; eight were composed of a child with EPP and healthy parents, one has two affected siblings, and one family has a son and mother with EPP. When analyzing the previously described candidate variants associated with pituitary development, we found variants in *GLI2* and *FGFR1* in three families. We also found six other variants of interest in three patients: *KMT2A*, *GALR3*, *RTN4R*, *SEMA3A*, *NIPBL*, and *DSCAML1*.

**Conclusion:**

The analysis allowed us to find previously reported and not reported *GLI2* variants, all inherited from healthy parents, which reinforces the incomplete penetrance pattern of *GLI2* variants in the development of EPP and draws attention to possible future functional studies of those variants that have a recurrent expression in CH. We also found novel *FGFR1* and *SEMA3A* variants that suggest an oligogenic mechanism in PSIS and EPP, as seen in patients with hypogonadotropic hypogonadism. We report the first case of a patient with Wiedemann-Steiner syndrome and PSIS, suggesting that the *KMT2A* gene may be related to pituitary development. Furthermore, the trios' analysis allowed us to find five other variants of interest. Future investigations may clarify the roles of these variants in the etiology of EPP and PSIS.

## 1 Introduction

Ectopic posterior pituitary (EPP) is a rare congenital malformation that occurs due to defective neuronal migration, in which the posterior pituitary is usually located outside its anatomical location in the sella turcica. The posterior lobe can usually be identified at the base of the third ventricle by a hyperintense signal on T1-weighted magnetic resonance imaging (MRI) in patients with EPP (1). It may be associated with other midline malformations, such as the absence or thinning of the pituitary stalk and empty pituitary fossa, in this case being referred to as pituitary stalk interruption syndrome (PSIS) (2). Patients with EPP have pituitary dysfunction, commonly presenting growth hormone deficiency (GHD) in isolation or associated with multiple pituitary hormone deficiencies (MPHD) (3).

Most congenital hypopituitarism (CH) cases are sporadic, with few reports of familial cases. Some genes have been linked to pituitary embryonic development (4, 5).

Genes such as *OTX2*, *LHX4*, *HESX1*, *SOX3*, and *GLI2* responsible for pituitary embryonic development seem to play a role in midline congenital malformations, including EPP. However, about 90% of CH cases remain unsolved (6). Other genes related to EPP and PSIS likely remain to be discovered. Therefore, we performed trio-based whole-exome sequencing (WES) on patients with EPP and their families, including two familial cases, to search for candidate genes.

## 2 Methods

This study included subjects from a Pediatric Endocrinology Unit of a tertiary Hospital diagnosed with EPP by a simplified FAST1.2-MRI protocol (1, 7). This protocol does not employ sedation, anesthesia, or contrast. It includes a sagittal-T1 sequence acquisition and an additional 3D T2DRIVE sequence, and the whole imaging acquisition lasts about 7 min. FAST1.2-MRI was also performed to exclude EPP in first-degree relatives of patients. The following HP abnormalities were described: anterior pituitary (hypoplastic/normal), ectopic posterior pituitary, and pituitary stalk (absent/thin/normal). We chose this approach considering the lack of universally accepted reference standards for the size of the hypothalamic-pituitary (HP) structures in the pediatric age; the data collected were qualitative and descriptive. When reporting distinctive facial features, we did not include the facial dysmorphisms commonly observed in GHD (frontal bossing, saddle nose, and maxillary hypoplasia); we included only features that could be related to the potential underlying pathogenic variant.

Clinical data, gestational and birth history data were collected. Height standard deviation score (SDS), weight, and BMI SDS were measured (8). Birth length and weight were evaluated (9).

Laboratory tests were performed to confirm hypopituitarism. GH deficiency was confirmed by low IGF-1 levels associated with low growth velocity <−2SDS for age and sex and/or short stature (height <−2SDS), the presence of MPHD and EPP, or by a GH peak response of less than 5 ng/ml in two stimulation tests (insulin and clonidine or glucagon tests). However, it is important to reinforce that some patients were younger than two years old and had low weight, which made the stimuli test unfeasible, and therefore the first criteria were adopted (10). Besides, one patient (Patient 5), although she was older than two years, did not perform GH stimulation test because she already had multiple hormone deficiencies, low levels of IGF1, short stature (SDS −2.96), and a malformation of CNS compatible with hypopituitarism. Our group previously reported a High Frequency of Normal Response during GH Stimulation Tests in Patients with EPP; one of the patients fits these criteria (11). Central hypothyroidism was defined as low free thyroxine (F-T4) values associated with low/normal thyroid-stimulating hormone (TSH) values. ACTH-dependent cortisol insufficiency was defined as 8:00 a.m. cortisol values lower than 5mcg/dl and low/normal levels of adrenocorticotropic hormone (ACTH) (12) Hypogonadotropic hypogonadism was considered only in patients who had reached the pubertal age as the absence of thelarche in girls after 13 years of age and no testicular volume increase in boys after 14 years of age, associated with low gonadotropins, estradiol in girls or total testosterone in boys. The presence of micropenis and cryptorchidism were also considered indicative of central hypogonadism. Micropenis was defined as a penile size 2.5 SDS below the mean for age (13).

We collected blood samples according to routine follow-ups of patients and their parents; DNA was extracted from peripheral blood leukocytes (14). DNA samples were submitted to WES, employing Illumina technology Alignment and identification of variants using standard bioinformatics protocols, having as reference the Genome Reference Consortium Human Build 38 of the human genome. We identified and classified variants of clinical relevance using the Franklin platform (https://franklin.genoox.com/clinical-db/home).

The medical analysis was guided by the detailed clinical picture of each patient, including their phenotype and hormonal deficiencies. Our analysis was limited to five criteria: (1) rare variants with a minor allele frequency (AF) less than 0.01 in two public genomic databases: the Genome Aggregation Database (gnomAD) and the Online Archive of Brazilian Mutations (ABraOM). (2) Sequencing coverage >10 reads. (3) Altered the predicted amino acid sequence of the encoded protein (i.e., missense, nonsense, frameshift/non-frameshift insertions or deletions, and splicing variants). (4) Predicted pathogenic by Revel score. (5) Probability of being loss-of-function (LoF) intolerant score (pLI) ≥0.9. 6) Confirmed to be present by visual inspection of Binary Alignment Map (BAM) files. All possible candidate variants were classified according to the American College of Medical Genetics and Genomics (ACMG) standards (15).

We performed two distinct analyses in the trio-based WES. First, we looked for 66 previously described candidate gene variants associated with pituitary development (Supplementary Table S1) (6, 15, 16), regardless of inheritance pattern, to broadly screen for any genetic contribution, including incomplete penetrance and oligogenic causes. Next, we investigated the whole exome to search for variants inherited in a pattern consistent with a monogenic etiology.

The analysis of the WES data was carried out in families that had an affected proband and two unaffected parents (families 3–10) in search of rare variants present in the proband and absent in their parents, with the presumption of dealing with an autosomal dominant disease resulting from a “*de novo*” mutational event in the proband. We also looked for rare variants in homozygosity or compound heterozygosity in the proband to consider the hypothesis of a condition with an autosomal recessive inheritance pattern. In male patients, rare variants in hemizygosity on the X chromosome were also evaluated, considering the possibility of a disease with X-linked inheritance.

Family 1 consists of two male siblings with EPP. We performed WES to investigate possible variants associated with EPP in both siblings. We searched for rare variants in homozygosity or compound heterozygosis present in both siblings (autosomal recessive). In addition, we also looked for hemizygous variants in X chromosome genes shared by the two brothers (X-linked). Additionally, we investigated the possibility of rare heterozygous variants present in both EPP carriers and absent in their parents to consider a *de novo* autosomal dominant disease.

Family 2 consists of a son and a mother with EPP. The mother with EPP has short stature (Height of 151.5 cm), normal puberty, and at the moment, she is overweight. She has had no other hormonal deficiency detected until now. We searched for variants that affected the proband and his mother, with the presumption of dealing with an autosomal dominant disease. We also looked for rare variants in homozygosity or compound heterozygosity present in both (autosomal recessive) and X-linked dominant variants.

The Ethics Committee approved the study protocol (CAAE: 85543918.0.0000.5479). Written informed consent was obtained from the patient's guardians. Data were prospectively recorded in the Research Electronic Data Capture (REDCap) database.

The following inclusion/exclusion criteria were applied:

Inclusion criteria: patients with short stature and MRI with EPP associated or not with adenohypophysis hypoplasia (<0.3 cm) and pituitary stalk thinning (<0.2 cm - measured horizontally in the median portion of the stalk) or absence, with at least two family members who agreed to undergo MRI and blood collection.
Exclusion criteria: patients that could have other causes for hypopituitarism (ex: associated traumatic brain injury) or a diagnosis of other complex central nervous system malformation, including septo-optic dysplasia (SOD) and holoprosencephaly.

Statistical analysis was performed using SigmaStat 3.5 for Windows (SPSS, Point Richmond, CA, USA). We used a *Z*-test to compare the proportion of findings between groups. A *p*-value of <0.05 and a 95% confidence interval were considered statistically significant.

## 3 Results

**Figure 1:**
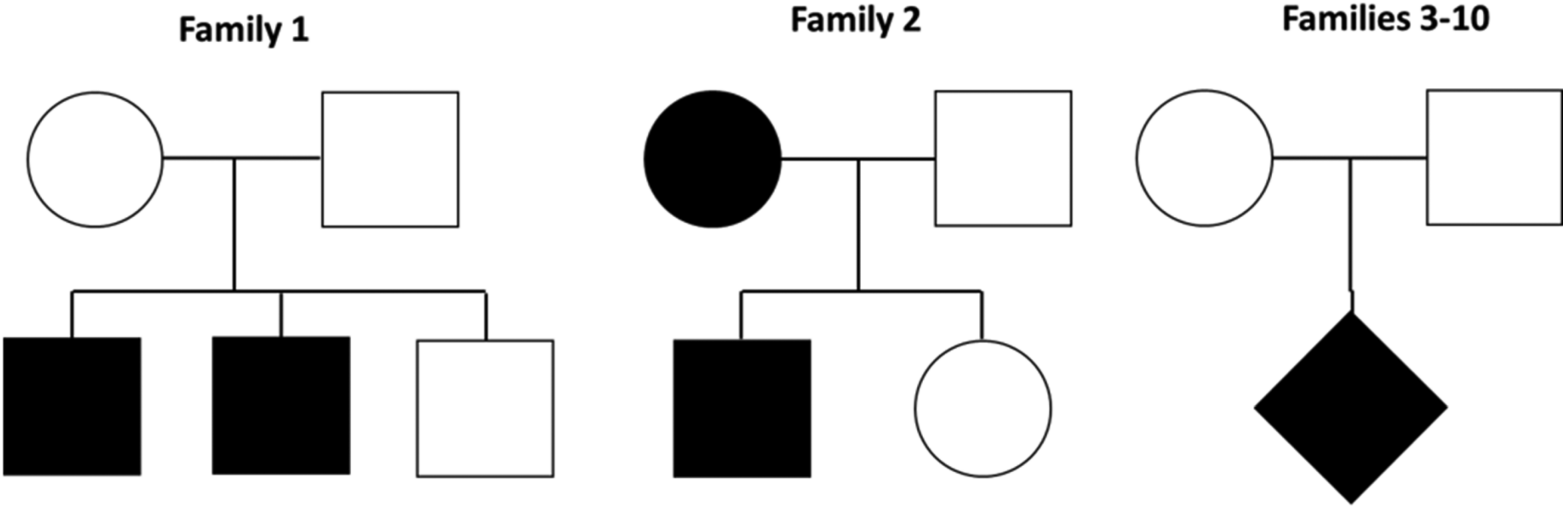
Families evaluated by exomic sequencing. Family 1 has two affected siblings, family 2 a son and a mother with EPP and families 3–10 a child with EPP and healthy parents.

Ten families with EPP were evaluated; eight were composed of a child with EPP and healthy parents, one has two affected siblings, and another family has a son and mother with EPP (Figure 1). None of the families have a history of consanguinity.

Height, weight, BMI, hormonal profile, MRI findings, associated disease and malformations, and pregnancy and birth data from the moment of initial diagnosis of EPP are detailed in Table 1. As EPP may initially present with GHD and progress to MPHD, some of the values in Table 1 may not reflect laboratory results from the diagnosis of new pituitary deficiencies. Four (36.4%) children have only GHD. Six (54.5%) patients were diagnosed with PSIS (anterior pituitary hypoplasia, thin or absent pituitary stalk, and EPP). We observed MPHD in 5/6 patients with PSIS and 2/5 with isolated EPP (83.3% vs. 40%, *Z*-test; *p* = 0.391). Patients with MPHD were younger at diagnosis, with a mean age (SD) of 3.1 (3.2) than those with isolated GHD 9.2 (3.8) (*p* = 0.017). One patient was born with a length less than −2 SDS. None of the cases had a birth weight less than −2 SDS (1) (Table 1).

**Table 1:** Anthropometric, hormonal and MRI data at diagnosis and in the neonatal period of the 11 patients with ectopic posterior pituitary (EPP) and pituitary stalk interruption syndrome (PSIS).

Family	1.1	1.2	2	3	4	5	6	7	8	9	10
Gender	Male	Male	Male	Male	Male	Female	Male	Male	Female	Male	Female
Age years	10.6	14.5	5.8	7.2	8.6	3.5	0.2	1.2	1.1	1	6.6
Height cm (SDS)	121.2 (−3.09)	132 (−4.58)	95 (−4.17)	108.8 (−2.06)	115.8 (−2.54)	87 (−2.96)	51.0 (−3.3)	73 (−1.8)	61.5 (−4.9)	70.1 (−2.0)	99.8 (−5.96)
Weight kg	22.3	28.6	14.6	18.1	26.7	12.5	3.6	10.2	5.9	6.9	15.8
BMI (SDS)	15.2 (−1.02)	16.4 (−1.64)	16.2 (+0.63)	15.3 (−0.19)	19.9 (+1.91)	16.5 (+0.84)	13.7 (−2.0)	18.8 (+1.5)	15.6 (−0.7)	13.8 (−2.6)	16.5 (+0.67)
GH peak (ng/ml)	9.2	1.1	2.5	1.4	1.7	Not tested	Not tested	Not tested	Not tested	Not tested	0.7
IGF-1 (ng/mL)/(SDS)	82 (−2)	96.8 (−2.3)	25 (−2.3)	48.4 (−2.4)	<15 (−2.7)	<25 (−2.1)	66 (−1.6)	55(−1.8)	23 (−2.3)	<15 (−2.2)	<25 (−2.5)
Cortisol (µg/dl)	12	17.7	7.7	9.3	19.5	6.4	<0.1	1.4	1.6	0.9	2.9
Free T4 (ng/dl)	1.2	1.3	0.9	0.9	0.7	0.9	0.7	0.6	1.1	0.8	0.9
TSH (µUI/ml)	1.09	3.63	2.1	2.07	2.95	0.53	2	4.1	2.67	3.9	4.86
Hormonal deficiencies	GH	GH	GH	GH	GH, TSH	GH, TSH, ACTH	GH, ACTH	GH, TSH, ACTH	GH, ACTH	GH, TSH, ACTH	GH, TSH, ACTH, LH/FSH^a^
MRI findings	AP normal EPP-ME PS thin	AP normal EPP-ME PS thin	AP hypoplastic EPP-ME PS normal	AP hypoplastic EPP-ME PS thin	AP normal EPP-ME PS thin	AP hypoplastic EPP-ME PS thin	AP hypoplastic EPP-ME PS absent	AP hypoplastic EPP-ME PS absent	AP hypoplastic EPP-ME PS absent	AP normal EPP-ME PS normal	AP hypoplastic EPP-ME PS absent
Associated diseases and malformations	None	None	None	None	Congenital clubfoot	None	Patent foramen ovale, low-set ears, bifid nasal tip	Micropenis, patent foramen ovale	Bilateral hydronephrosis, low-set ears	Ear appendix	None
Target height cm (SDS)	168 (−1.1)	168 (−1.1)	165.5 (−1.5)	163 (−1.8)	167 (−1.3)	160 (−0.5)	177.5 (0.2)	181 (0.7)	167.3 (0.6)	177.5 (0.2)	170 (1.1)
Pregnancy complications	None	None	None	premature labor	None	Use of misoprostol and tobacco	Hypertensive disease of pregnancy	None	None	Mother with hypothyroidism	None
Birth type	Vaginal, cephalic	Vaginl, cephalic	C-section, cephalic	Vaginal, cephalic	C-section, cephalic	C-section, cephalic	C-section, cephalic	C-section, cephalic	C-section, cephalic	C-section, cephalic	C-section, cephalic
Gestational age	39	40	39	34 + 2/7	39 + 1/7	40	39 + 5/7	38 + 2/7	38 + 1/7	40 + 4/7	39
Birth length cm (SDS)	47 (−1.41)	51 (−0.07)	47 (−1.41)	50 (+1.96)	49 (−0.6)	47 (−1.85)	46 (−2.17)	47 (−1.10)	45 (−1.89)	51 (−0.31)	49 (−0.54)
Birth weight g (SDS)	2,800 (−1.27)	3,850 (+0.59)	3,310 (−0.11)	3,340 (+2.5)	3,540 (+0.32)	3,410 (−0.34)	3,075 (−0.96)	3,250 (+0.08)	3,900 (+1.53)	3,715 (+0.05)	3,015 (−0.77)
Neonatal complications	None	None	None	Jaundice and hypoglycemia	Jaundice	None	Hypoglycemia	Jaundice, hypoglicemia and respiratory distress	Jaundice and hypoglycemia	None	Jaundice

SDS, standard deviation score; BMI, body mass index; MRI, magnetic resonance imaging; AP, anterior pituitary; EPP in median eminence (EPP-ME).

^a^ Patient 10 had her puberty induced at 14 years old and currently (at 17 years old) she is on estrogen and progesterone replacement.

We found potential variants in two genes in three families when analyzing the previously described candidate variants associated with pituitary development (Table 2). We found a *GLI2* variant in both siblings from family 1, inherited from the mother. In family 7, the proband carried an *FGFR1* missense variant inherited from his mother. Proband from family 10 carries two *GLI2* variants inherited from her mother and one from her father.

**Table 2:** Families evaluated by whole exome sequencing (WES).

Family/Proband	Gene	Inherited from	Aminio acid change	Nucleotide	dbSNP	Transcript	Effect	gnomAD MAF	ABraOM MAF	Revel	LOF homozygotes	Franklin ACGM
1	*GLI2* ^a^	Mother	p.Ala268Val	c.803C>T	rs146992756	NM_001374353.1	Missense	0.08%	0.4%	Uncertain (0.40)	N/A	Benign
7	*FGFR1*	Mother	p.Ala36Pro	c.106G>C	N/A	NM_023110.3	Missense	N/A	N/A	Uncertain (0.3)	N/A	VUS
10	*GLI2* ^a^	Mother	p.Leu1428Phe	c.4282C>T	rs146207623	NM_001374353.1	Missense	0.91%	0.73%	Benign (0.07)	N/A	Benign
10	*GLI2* ^a^	Mother	p.Met1427Ile	c.4281G>A	rs146467786	NM_001374353.1	Missense	0.91%	0.73%	Benign (0.03)	N/A	Benign
10	*GLI2*	Father	p.Ala200Thr	c.598G>A	rs111840592	NM_001374353.1	Missense	0.02%	N/A	Benign (0.17)	N/A	Likely Benign
10	*CDK5*	Father	p.Arg217^a^	c.649C>T	N/A	NM_004935.4	Stop gain	N/A	N/A	N/A	N/A	Likely pathogenic
3	*MAP1A*	Mother	p.Pro2394Leu	c.7181C>T	rs749548952	NM_002373.6	Missense	<0.01%	N/A	Benign (Moderate) (0.03)	N/A	VUS
3	*MAP1A*	Father	p.Pro1951Leu	c.5852C>T	rs768903001	NM_002373.6	Missense	<0.01%	N/A	Benign (0.07)	N/A	VUS
3	*PROK2*	Father	p.Gly100fs	c.297dupT	rs768413190	NM_001126128.2	Frameshift	0.01%	N/A	N/A	N/A	Likely pathogenic
5	*GALR3*		p.Arg120Trp	c.358A>T	rs1373587952	NM_003614.2	Missense	<0.01%	N/A	Deleterious (Strong) (0.98)	N/A	Likely pathogenic
8	*KMT2A*		p.Cys1155ValfsTer20	c.3462delG	N/A	NM_001197104.2	Frameshift	N/A	N/A	N/A	N/A	Pathogenic
8	*RTN4R*		p.Leu249fs	c.745delC	N/A	NM_023004.6	Frameshift	N/A	N/A	N/A	N/A	VUS
8	*SEMA3A*	Mother	p.Arg484Trp	c.1450C>T	rs137871935	NM_006080.3	Missense	0.018%	N/A	Benign (0.23)	N/A	VUS
9	*NIPBL*		p.Gln1496Lys	c.4486C>A	N/A	NM_133433.4	Missense	N/A	N/A	Deleterious (0.87)	N/A	VUS
9	*DSCAML1*		p.Ala675Ser	c.2023G>T	N/A	NM_020693.4	Missense	N/A	N/A	Uncertain (0.42)	N/A	VUS

Description of genetic variants associated with pituitary development and candidate variants for a monogenic etiology. MAF, minor allele frequency; N/A, not available.

^a^ Variants previously described in patients with hypopituitarism.

We also found six other variants of interest in three patients in the investigation of the whole exome (Table 2). In proband 3, we found a compound heterozygous variant in *MAP1A*, probably benign. Patient 5 had a *de novo* probably pathogenic variant in *GALR3* gene. Proband 8 carried a *SEMA3A* missense variant inherited from her mother and a *de novo* variant in *KMT2A* and *RTN4R* genes. In family 9, we found *de novo* variants in two genes: *NIPBL* and *DSCAML1*. In Family 2, no pathogenic genetic variants that justify the clinical manifestations in the proband and his mother were found. No pathogenic genetic variants were identified in families 4, 6, and 7.

## 4 Discussion

For a rare disease like EPP, this is an extensive study of trios submitted to WES. Furthermore, we describe two familial cases. The patients included were phenotypically non-syndromic and had no severe central nervous system (CNS) malformations with the aim of looking for genes that could clarify the etiology of EPP and PSIS as individual pathologies, not as part of syndromes like septo-optic dysplasia (SOD) and holoprosencephaly.

One patient was born with a length less than −2 SDS. However, this patient had a history of a hypertensive disease of pregnancy, which is a maternal cause related to intrauterine growth restriction. None of the subjects had a birth weight less than −2 SDS. This data agrees with the literature that patients with CH have normal weight and length at birth (17).

In family 1, we found a *GLI2* variant present in both siblings and inherited from their mother. Proband from family 10 also carries two *GLI2* variants inherited from her mother and one from her father. *GLI2* is a transcription factor involved in the Sonic Hedgehog pathway. *GLI2* pathogenic variants are known to cause defects in neuronal differentiation, proliferation, and migration associated with MPHD (6). The variants in family 1 and both variants inherited from the mother in family 10 (Table 2) are found with a frequency of more than 0.01 in databases and they were considered benign and probably unrelated to the EPP phenotype; however, those variants were previously described in patients with EPP. Arnhold et al. published a review of the evidence for *GLI2* mutations as a cause of hypopituitarism. They concluded that there is a relatively high frequency of *GLI2* variants in patients with GHD and EPP and that *GLI2* might interact with other epigenetic factors to modulate the phenotype in an incomplete penetrance pattern (18). Although it is unclear if those variants have functional significance, we believe it is important to describe them to contribute to the number of reports of common variants in cohorts with hypopituitarism, thus drawing attention to possible future studies of variants with recurrent expression in this population.

In proband 3, we found a compound heterozygous variant in *MAP1A* (Table 2). *MAP1A* is expressed almost exclusively in the brain and belongs to a family involved in microtubule assembly, a crucial step in neurogenesis. Protein-truncated variants in *MAP1A* were recently related to the presence of autism spectrum disorder (ASD) and attention-deficit/hyperactivity disorder (ADHD) in a large cohort (19). However, the patient studied does not present symptoms compatible with these conditions and based on the REVEL analysis, both variants (maternal and paternal) are benign, so we did not considered it as a causative of PSIS in this case.

In patient 5, we found a *de novo* vartiant in *GALR3* gene, probably pathogenic (Table 2). *GALR3* gene encodes galanin receptor type 3 and this receptor is a member of the G protein-coupled receptor. Its main known ligand is galanin, but not all the ligands to GLR3 and its biological effects signalization are still not fully understood. Galanin is a neuropeptide, expressed in many tissues, specially in central nervous system and it is associated with a lot of central and peripheral receptor-mediated actions including feeding and anterior pituitary hormone regulation (20). Although no association between pathogenic variants in *GALR3* gene and EPP has been described until now, we think that this gene could be of interest in this disease.

Proband 7 carried an *FGFR1* missense variant. In addition, *FGFR1* variants were also reported in patients with PSIS and SOD. Correa et al. reported a deleterious *FGFR1* variant in a PSIS patient and her unaffected mother; this variant was submitted to functional analysis that showed a reduced signaling activity, reinforcing that this was likely contributing to the phenotype, although first-degree relatives were unaffected carriers (21). Our case 7 also has PSIS, and his *FGFR1* variant is also inherited from his mother. However, in our study this variant was considered probably not responsible for the phenotype.

In patient 8, we found a pathogenic variant in the *KMT2A* gene (according to ACMG criteria). Loss of function variants (LoF) in the *KMT2A* gene are associated with Wiedemann-Steiner syndrome (WSS, OMIM#605130). WSS is characterized by facial dysmorphism, hypertrichosis of the elbow, psychomotor delay, and short stature that can be caused by GHD. We found a report by Stoyle et al. (22) of a patient with WSS with a minor posterior pituitary ectopia with elongation into the lower part of the pituitary stalk (22). The *KMT2A* is essential for properly developing brain architecture, as LoF and animal knock-out models show premature neuronal differentiation (23). Patient 8 has a mild phenotype, with bilateral hydronephrosis and low-set ears, both associated with WSS (24). To our knowledge, this is the first report of a WSS associated with PSIS. Those findings suggest that *KMT2A* may have a role in pituitary development. EPP and PSIS are not described as typical features of WSS, and we believe that those characteristics are underdiagnosed due to a lack of pituitary MRI investigation, even in patients with confirmed GHD. We also believe that FAST1.2 MRI protocols could help detect cases of EPP or PSIS, as it is a fast and simple protocol with no need for contrast (1, 7).

Proband 8 also carried a *SEMA3A* missense variant inherited from her mother. *SEMA3A* is a member of the semaphorin family and is vital for normal neuronal pattern development. It was described in patients with anosmic hypogonadotropic hypogonadism. However, based on the seemingly normal reproductive phenotype of Sema3a ± mice, Hanchate et al. suggested that monoallelic pathogenic variants in *SEMA3A* are not sufficient to induce the abnormal phenotype in patients but contribute to the pathogenesis of HH through synergistic effects with mutant alleles of other disease-associated genes. Hanchate et al. (25) described 24 patients with heterozygous *SEMA3A* variants; interestingly, five of those patients had a previous diagnosis of HH caused by pathogenic variants in genes such as *PROKR2*, *PROK2*, *FGFR1*, and *KAL1* (25). It is known that variants in the *FGFR1* gene are related not only to HH but also to SOD and PSIS (6, 21, 25, 26). This draws our attention to whether *SEMA3A* could also be related to an oligogenic pattern of pituitary development related to EPP and PSIS. We also found a *de novo* variant in the *RTN4R* gene in this patient. *RTN4R* is highly expressed in the CNS and mediates axonal growth inhibition, regeneration, and plasticity (27).

In family 9, we found *de novo* variants in two genes: *NIPBL* and *DSCAML1*. *NIPBL* missense variants are related to Cornelia de Lange syndrome (CdLS). CdLS has a broad spectrum of clinical involvement characterized by dysmorphic facial features, which are absent in the patient (Table 1). Studies in zebrafish with *NIPBL* knockdown show downregulation of canonical *WNT*-pathway genes and impaired neural development (28). It has been proven that the WNT/B-catenin signaling pathway participates in the proliferation of Rathke's pouch and differentiation in pituitary cells (6, 23). The p.Gln1496Lys variant has not been described to date in population databases and may be the first report of a patient carrying a *NIPBL* variant and EPP. *DSCAML1* is highly expressed in the brain, including the hypothalamus and pituitary, and is involved in neuronal migration and positioning. Recently, the relationship of heterozygous variants in the *DSCAML1* gene with epilepsy and neurodevelopmental disorders (NND) has been studied (29).

The remaining families (2, 4, 6, and 7) submitted for WES in our study had no potential pathogenic variant. Most patients with hypopituitarism remain without a molecular diagnosis, and whether local mosaicisms could cause malformations of hypothalamic-pituitary structures is questioned. Another challenge of molecular diagnosis in hypopituitarism is the presence of variants with incomplete penetrance, as reported in two families with *GLI2* variants in this study (15, 25). Furthermore, there is suspicion that some cases could be related to digenic or oligogenic patterns, as described in the *SEMA3A* variants in HH, in which variants present in isolated genes sometimes do not cause the phenotype; however, combined, they can cause the disease. Alternatively, the condition could be caused by a combined effect of pathogenic variants in addition to environmental or epigenetic factors.

Although *MAP1A, RTN4R, NIPBL*, and *DSCAML1* variants have been shown to affect the brain adversely, no specific reports on EPP or other hypothalamus-pituitary region malformations have been published previously to our knowledge.

Our study had some important strengths. We evaluated WES in a significant population for rare diseases like EPP and PSIS, including two familial cases (families 1 and 2). Trios' analysis allowed the elimination of variants of uncertain significance (VUS) present in parents.

This study also has some limitations. WES does not target the non-coding portions of the genome, such as those in promoter regions, enhancers, or microRNAs. A further study exploring these regulatory regions may reveal additional important genetic cases of congenital hypopituitarism. Although the population was significant for a condition like EPP and PSIS, it was still small in terms of power to identify statistically significant associations. Despite functional analysis remains vital in determining the pathogenicity of all novel variants, we could not perform them in this study. We intentionally selected phenotypically non-syndromic patients without complex CNS malformations to search for genes that would justify the etiology of EPP and PSIS. This selection may have limited our findings since syndromic patients have the most potential to find new pathogenic variants. Also, unfortunately, no Sanger sequencing was made to check those variants due to a cost limitation. However, whether Sanger is still necessary as a verification method for high-quality single-nucleotide and small insertion/deletion variants has been debatable (30).

In conclusion, we performed WES in a significant population for rare diseases like EPP and PSIS. The analysis of previously described candidate gene variants associated with pituitary development allowed us to find three *GLI2* variants previously reported in patients with EPP and one variant with no prior description, all inherited from healthy parents, reinforcing the incomplete penetrance pattern of *GLI2* variants in the development of EPP, and drawing attention to possible future functional studies of those variants that have recurrent expression in this population. We found novel *FGFR1* and *SEMA3A* variants that suggest the possibility of an oligogenic mechanism in PSIS and EPP, as seen in patients with HH. We report the first case of a patient with WSS and PSIS, suggesting that the *KMT2A* gene may be related to pituitary development. Furthermore, trios' analysis allowed us to find five other variants associated with neuronal development. Future investigations may clarify the roles of these variants in the etiology of EPP and PSIS.

## Data Availability

The data presented in the study are deposited here: https://www.ncbi.nlm.nih.gov/bioproject/1136988, accession number: RJNA1136988.

## Supplementary material

Supplementary Table S1 Genetic variants found in 66 described hypopituitarism-associated genes: ARID1B, ARNT2, BMP2, BMP4, CDON, CHD7, EIF2S3, FEZF1, FGF10, FGF18, FGF8, FGFR1, FOXA2, GATA2, GH1, GLI1, GLI2, GLI3, GLI4, GLI5, GLI6, GPR161, HESX1, IFT172, IGSF1, KAL1, KCNQ1, KMT2A, KMT2D, LHX3, LHX4, MAGEL2, NFKB2, NR5A1, OTX2, PAX6, PC1, PCSK1, PITX1, PITX2, PNPLA6, POMC, POU1F1, PROK2, PROKR2, PROP1, PTCH1, RAX, RNPC3, ROBO1, SHH, SIX1, SIX2, SIX3, SIX4, SIX5, SIX6, SOX1, SOX2, SOX3, TC1D32, TBX19, TCF7L1, TGIF, UBR1, and ZIC2.
