# Supplementary material for: Trio-based whole exome sequencing in patients with ectopic posterior pituitary
Source: Front Pediatr. 2024 Aug 2;12:1334610. doi: 10.3389/fped.2024.1334610 (PMC11327137; doi:10.3389/fped.2024.1334610)
Supplement: Supplementary Table S1 — Genetic variants found in 66 described hypopituitarism-associated genes: ARID1B, ARNT2, BMP2, BMP4, CDON, CHD7, EIF2S3, FEZF1, FGF10, FGF18, FGF8, FGFR1, FOXA2, GATA2, GH1, GLI1, GLI2, GLI3, GLI4, GLI5, GLI6, GPR161, HESX1, IFT172, IGSF1, KAL1, KCNQ1, KMT2A, KMT2D, LHX3, LHX4, MAGEL2, NFKB2, NR5A1, OTX2, PAX6, PC1, PCSK1, PITX1, PITX2, PNPLA6, POMC, POU1F1, PROK2, PROKR2, PROP1, PTCH1, RAX, RNPC3, ROBO1, SHH, SIX1, SIX2, SIX3, SIX4, SIX5, SIX6, SOX1, SOX2, SOX3, TC1D32, TBX19, TCF7L1, TGIF, UBR1, and ZIC2. [file Table1.pdf]

| Family/ Proband | Gene | Inherited from | Aminio Acid Change | Nucleotide | dbSNP | Transcript | Effect | gnomAD MAF | ABraOM MAF | Revel | LOF Homozygotes | Franklin ACGM |
|---|---|---|---|---|---|---|---|---|---|---|---|---|
| 1 | *GLI2** | Mother | p.Ala268Val | c.803C>T | rs146992756 | NM_001374353.1 | Missense | 0.08% | 0.4% | Uncertain (0.40) | N/A | Benign |
| 7 | *FGFR1* | Mother | p.Ala36Pro | c.106G>C | N/A | NM_023110.3 | Missense | N/A | N/A | Uncertain (0.3) | N/A | VUS |
| 10 | *GLI2** | Mother | p.Leu1428Phe | c.4282C>T | rs146207623 | NM_001374353.1 | Missense | 0.91% | 0.73% | Benign (0.07) | N/A | Benign |
| 10 | *GLI2** | Mother | p.Met1427Ile | c.4281G>A | rs146467786 | NM_001374353.1 | Missense | 0.91% | 0.73% | Benign (0.03) | N/A | Benign |
| 10 | *GLI2* | Father | p.Ala200Thr | c.598G>A | rs111840592 | NM_001374353.1 | Missense | 0.02% | N/A | Benign (0.17) | N/A | Likely Benign |
| 10 | *CDK5* | Father | p.Arg217* | c.649C>T | N/A | NM_004935.4 | Stop gain | N/A | N/A | N/A | N/A | Likely Pathogenic |
| 3 | *MAP1A* | Mother | p.Pro2394Leu | c.7181C>T | rs749548952 | NM_002373.6 | Missense | < 0.01% | N/A | Benign (Moderate) (0.03) | N/A | VUS |
| 3 | *MAP1A* | Father | p.Pro1951Leu | c.5852C>T | rs768903001 | NM_002373.6 | Missense | < 0.01% | N/A | Benign (0.07) | N/A | VUS |
| 3 | *PROK2* | Father | p.Gly100fs | c.297dupT | rs768413190 | NM_001126128.2 | Frameshift | 0.01% | N/A | N/A | N/A | Likely Pathogenic |
| 5 | *GALR3* | | p.Arg120Trp | c.358A>T | rs1373587952 | NM_003614.2 | Missense | < 0.01% | N/A | Deleterious (Strong) (0.98) | N/A | Likely Pathogenic |
| 8 | *KMT2A* | | p.Cys1155ValfsTer 20 | c.3462delG | N/A | NM_001197104.2 | Frameshift | N/A | N/A | N/A | N/A | Pathogenic |
| 8 | *RTN4R* | | p.Leu249fs | c.745delC | N/A | NM_023004.6 | Frameshift | N/A | N/A | N/A | N/A | VUS |
| 8 | *SEMA3A* | Mother | p.Arg484Trp | c.1450C>T | rs137871935 | NM_006080.3 | Missense | 0.018% | N/A | Benign (0.23) | N/A | VUS |
| 9 | *NIPBL* | | p.Gln1496Lys | c.4486C>A | N/A | NM_133433.4 | Missense | N/A | N/A | Deleterious (0.87) | N/A | VUS |
| 9 | *DSCAML1* | | p.Ala675Ser | c.2023G>T | N/A | NM_020693.4 | Missense | N/A | N/A | Uncertain (0.42) | N/A | VUS |

N/A: not available
